# Influence of Association on Binding of Disaccharides to YKL-39 and hHyal-1 Enzymes

**DOI:** 10.3390/ijms23147705

**Published:** 2022-07-12

**Authors:** Agnieszka Krzemińska, José-Emilio Sánchez-Aparicio, Jean-Didier Maréchal, Agata Paneth, Piotr Paneth

**Affiliations:** 1 International Center for Research on Innovative Biobased Materials (ICRI-BioM)-International Research Agenda, Lodz University of Technology, Żeromskiego 116, 90-924 Lodz, Poland; agnieszka.krzeminska-kowalska@p.lodz.pl; 2 Insilichem, Departament de Química, Facultat de Ciències, Universitat Autònoma de Barcelona, 08193 Cerdanyola del Vallès, Barcelona, Spain; joseemilio.sanchez@uab.cat; 3 Department of Organic Chemistry, Medical University of Lublin, Chodźki 4a, 20-093 Lublin, Poland; agata.paneth@umlub.pl

**Keywords:** disaccharides, complexation, docking, DFT, GaudiMM

## Abstract

Disaccharide complexes have been shown experimentally to be useful for drug delivery or as an antifouling surface biofilm, and are promising drug-encapsulation and delivery candidates. Although such complexes are intended for medical applications, to date no studies at the molecular level have been devoted to the influence of complexation on the enzymatic decomposition of polysaccharides. A theoretical approach to this problem has been hampered by the lack of a suitable computational tool for binding such non-covalent complexes to enzymes. Herein, we combine quantum-mechanical calculations of disaccharides complexes with a nonstandard docking GaudiMM engine that can perform such a task. Our results on four different complexes show that they are mostly stabilized by electrostatic interactions and hydrogen bonds. This strong non-covalent stabilization demonstrates the studied complexes are some excellent candidates for self-assembly smart materials, useful for drug encapsulation and delivery. Their advantage lies also in their biocompatible and biodegradable character.

## 1. Introduction

Advances in molecular-biology techniques and biotechnological processes have led to a plethora of therapeutical biomolecules with a high molecular weight that includes peptides, recombinant proteins, enzymes, monoclonal antibodies, antibody-drug conjugates, hormones, cytokines, blood factors, and nucleic acids [1]. Many of them require careful handling and storage conditions to ensure their quality. This is the case, for example, of thermal proteins with instability that requires storage and transport within a limited range of temperature to expand their lifetime. Humidity, photo exposure, oxygen, and mechanical agitation during transportation are other stress-related factors that impact stability. These therapeutics are also challenging in their delivery and bioavailability after oral intake. Most issues arise from (*i*) low drug solubility in physiological media; (*ii*) low drug stability in the physiological environment (i.e., drug degradation or denaturation); (*iii*) a substantial reduction in drug permeability across biological barriers such as skin, mucosal membranes, and cell membranes; (*iv*) incapability of the drug to passively reach its specific molecular target; and (*v*) the drug interacting with intracellular biomolecules before reaching its target. Currently, one approach consists of administrating these drugs through parenteral injections, although novel strategies are under vivid investigation and aim at enhancing stability, solubility, and bioavailability, while targeting specific tissues, cell populations, and/or the relevant subcellular structures.

One solution to the problems described above is to use polymeric nano- and microcapsules. Typically, capsular systems are made of a core–shell structure in which the inner core and the outer shell can be separately loaded with drug molecules or can be equipped with bio-functional ligands. As a smart material, such capsules are equipped with stimuli-responsive controlled-release functions for targeted and/or controlled release. These capsules are frequently constructed on saccharide motifs [2]. In this regard, efforts have been directed toward studying polysaccharides with repeating units that bear an electrolyte group (polyelectrolytes), such as chitosan (CHI) or furcellaran (FUR), or glycosaminoglycans such as hyaluronic acid (HA), cellobiose (CB), or heparine (HP). Such oppositely charged polysaccharides form complexes by non-covalent bonding (in particular, electrostatic, hydrogen bonds, and/or hydrophobic forces) that create three-dimensional networks that are being applied to drug- and cell-delivery carriers, as tissue adhesives or scaffolds for tissue engineering [3]. For example, positively charged CHI and negatively charged polysaccharides form complexes that can be used as a functional hemostat or as a wound dressing [3]. CHI/FUR was successfully applied as the nano-capsule carrier and delivery system of doxorubicin [4].

How these multimeric constructs impact the enzymatic decomposition in cells and how/if they lead to inhibition or activation processes have not been addressed yet. Despite a remarkable amount of experimental work performed on polysaccharides, there is very little information that can be found in the literature on the molecular description of their influence on the enzymes involved in their metabolism. In lieu of experimental data on how this complexation changes enzymatic catalysis, we decided to resort to computational tools to study these phenomena.

Gaining insight into these processes from a modeling perspective does not lack challenges though. First, no crystallographic structures have ever been reported in which multiple saccharides interacting between them are bound to a protein. This is, however, a prerequisite for starting the study we are aiming for. Second, the strength of the interactions between multiple saccharides could not be easily scored with standard force field approaches, so one needs some non-biased energetic methods such as quantum-mechanical ones (e.g., DFT) to estimate multi-saccharides affinities. Third, to get the initial models of complexes between multi-saccharides and protein receptors, protein–ligand dockings able to deal with multiple non-covalently bound ligands are necessary. Unfortunately, only a few programs allow multiple simultaneous bindings [5], although, to our knowledge, in all of them the ligands are considered as separated entities with no pre-existing non-covalent interaction networks. One way to tackle this problem is to apply GaudiMM [6], a multi-objective genetic algorithm that allows for guiding the conformational search towards complex solutions via additional molecular descriptors than the sole scoring function. For example, for the simultaneous docking of two ligands inside a protein host, one can consider the docking scoring function of the ligand complex together with additional evaluations of intermolecular (i.e., complex-protein) hydrogen-bond networks. Therefore, the combination of GaudiMM with other approaches such as quantum-mechanical calculations offers a unique framework to pursue our goal: learning how the complexation of different disaccharides influences their ability to bind to the enzymes responsible for their metabolic decomposition.

In this study, we have selected four complexes of disaccharides as models. This selection is based on (1) a variety of interacting charged groups, (2) the different net charges of the complexes, and (3) a different number of hydrogen bonds that exist in the complexes. In particular, we have studied the CHI/FUR complex binding to human cartilage chitinase 3-like Protein 2 (YKL-39), an enzyme that hydrolyzes the glycosidic bond in chitin. Bearing in mind that other polyelectrolytes, such as HA, HP, or CB, assembled in strongly bounded complexes can form nano-capsules, we have studied the interaction of HA/CHI, HA/CB, and HP/CB with lysosomal human hyaluronidase-1 (hHyal-1). This enzyme is responsible for HA hydrolysis and can be easily tracked experimentally since it can be detected in plasma and urine [7,8,9]. The basic properties of individual disaccharides and their complexes studied herein are summarized in Table 1.

## 2. Results and Discussion

### 2.1. Characterization of Interactions in Polyelectrolyte Complexes

**Figure 1:**
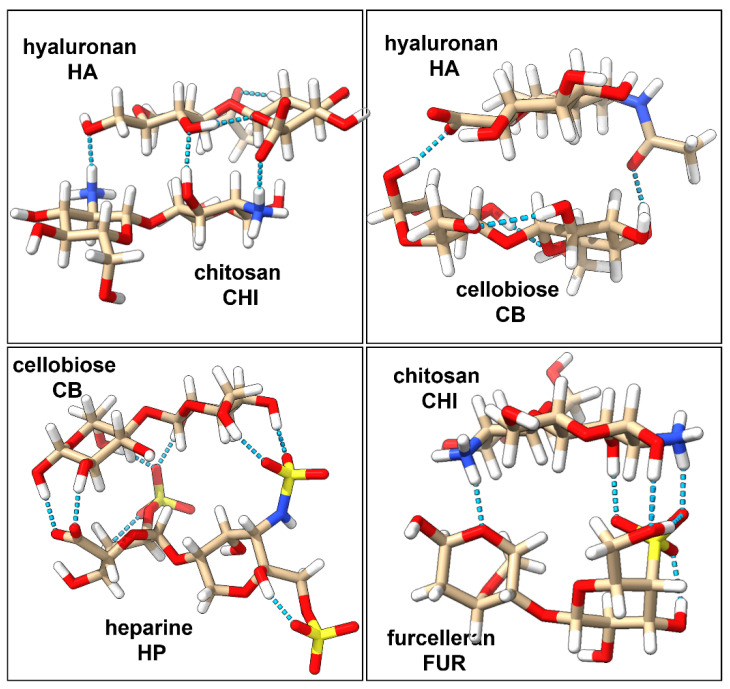
Optimized 3-dimensional structures of polyelectrolyte complexes of HA/CHI, HA/CB, HP/CB, and CHI/FUR with their key hydrogen bond interactions marked in light blue.

Interactions and strength of the four disaccharides complexes (HA/CHI, HA/CB, HP/CB, and CHI/FUR) have been characterized at the quantum level. The three-dimensional structures of the complexes are stabilized by non-covalent interactions, which can be strong or weak depending on changes in their charge distribution upon complexation [10]. Usually, it is a challenging task to evaluate the strength of the polyelectrolyte complexation energy because it depends on many environmental factors such as pH, ionic strength of the solution, the salt content, type of polyelectrolytes, or the presence of other charged entities [11]. In the literature, there are not many theoretical results that describe the strength of the polyelectrolyte complexes. Key hydrogen bond interactions identified in our studies are presented in Figure 1. Three inter-ligand hydrogen bonds in the HA/CHI complex have been identified: one between the amine-hydroxyl pair, one between the two hydroxyl groups, and one between the amine-carboxyl pair. In the HA/CB complex, two inter-ligand hydrogen bonds were identified: one between the carboxyl and hydroxyl groups and the other between the hydroxyl group and the oxygen from the amide group. The HP/CB complex has the most favorable hydrogen bond interaction network, due to the six hydrogen bonds between the hydroxyl groups from CB and the sulfate groups from HP, and an additional two hydrogen bonds between the hydroxyl groups of CB and the one carboxyl group of HP. Finally, the CHI/FUR complex contains four inter-ligand hydrogen bonds: one between the oxygen atom from the galactose ring of FUR and an amine group of CHI, one hydroxyl-pair hydrogen bond, one hydrogen bond between an amine group of CHI and the sulfate group of FUR, and one hydrogen bond between the hydroxyl group of CHI and the sulfate group of FUR.

The interaction energy for complex formation (Table 2) has been decomposed into the electrostatic, exchange, induction, and dispersion components. The HA/CB complex presents the lower formation energy (−43.9 kcal·mol^−1^), while the HP/CB complex presents the strongest one (−116.0 kcal·mol^−1^). Noticeably, those complexes are, respectively, with the lowest and highest amount of hydrogen bonds, therefore supporting the importance of such interactions in the stabilization of the complex. The other two complexes, HA/CHI and CHI/FUR, have intermediate energies of −109.2 kcal·mol^−1^ and −71.3 kcal·mol^−1^, respectively. Electrostatic attraction plays a dominant role in HP/CB formation and consists of 38.3% of the overall complexation energy, which exceeds the effects reflected in second-ordered terms (dispersion and induction). In the other three complexes, the electrostatic is about 1/3 of the total energy. Furthermore, the electrostatic attraction is larger for the CHI/FUR complex than the sum of the induction and dispersion terms, while for HA/CHI and HA/CB the electrostatic attraction has a similar value to second-ordered terms. Inductive attraction forces are equally important for HA/CHI (16.3%) and HP/CB (16.5%) formation, a little bit less for the HA/CB (14.3%) complex, and the lowest for CHI/FUR (12.8%). Surprisingly, a large dispersion contribution to the total complexation energy was computed for the HA/CB complex (18.4%) in contrast to the other complexes, where dispersion is only about 10%. For complexes characterized by strong attraction forces, it is not surprising that the repulsion exchange must be proportionately large, which is observed for the HA/CHI (45.4%) and CHI/FUR (46.7%) complexes. Relatively small repulsion was computed for the HA/CB (34.6%) and HP/CB (34.1%) complexes, perhaps due to the lack of the specific ionic nature of CB. The strongest electrostatic attraction and weakest exchange repulsion result in the strongest interaction energy obtained for the HP/CB complex.

### 2.2. Binding of Isolated Ligands and CHI/FUR Complex to YKL-39

**Figure 2:**
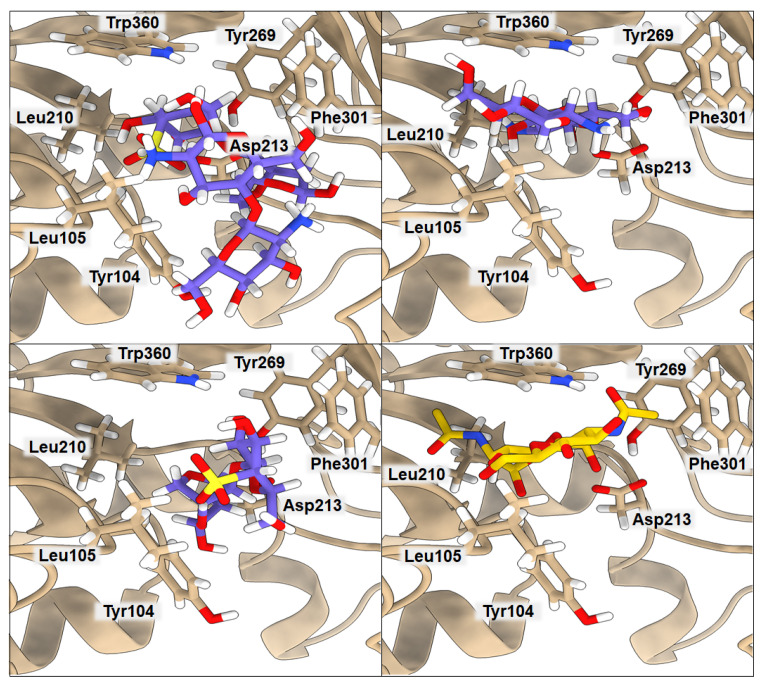
Ribbon representation of YKL-39 with docked CHI/FUR, CHI, FUR, and GlcNAc_2_ saccharides, respectively. Key amino acids that create interactions with ligands are marked in black.

According to Suginta and co-workers [12], chitooligosaccharide (GlcNAc_2_) binds to YKL-39 with hydrogen bonds and hydrophobic interactions, with Trp360, in the center of the chitin-binding cleft, playing a prominent role. It is believed that an arriving chitin chain would first attract the center of the binding site. The amino acids located in the center of the binding cleft make a highly ordered net of interactions, where Leu105 and Phe301 interact with one subsite of the ligand, and Tyr104, Asp213, Tyr269, and Trp360 stabilize the other part of the ligand. The binding affinity of GlcNAc_2_ to YKL-39, equal to −5.1 kcal·mol^−1^, was established by isothermal-titration calorimetry [12]. We used the crystal structure 4P8V deposited in the PDB by these authors for computing the binding affinity with PRODIGY. The obtained value of −5.7 kcal·mol^−1^ is in very good agreement with the experimental value, indicating the good performance of the PRODIGY methodology, which, in contrast to the free-energy-perturbation (FEP) and thermodynamics-integration (TI) methods, is less computationally time-consuming. Although using FEP or TI would probably lead to a more accurate binding-affinity value, the result obtained from PRODIGY should serve our purpose, since we want to compare several binding affinities for different ligands and systems, and we do not focus on absolute values. Results of the computed binding affinities of the GlcNAc_2_, FUR, CHI, and CHI/FUR complexes bound to the YKL-39 enzyme, along with a representation of the key interactions, are presented in Figure 2 and Table 3. In the case of the GlcNAc_2_ ligand, one of its subsites interacts through hydrogen bonds with Tyr104 (3.17 Å) and Asp213 (2.59 Å) and additional van der Waals interactions with Leu210 (4.08 Å) and Tyr269 (3.70 Å), plus it is stabilized by Trp360 (3.45 Å). The other subsite of the ligand interacts via van der Waals interactions with Leu105 (3.02 Å) and Phe301 (3.91 Å). Interestingly, both CHI and FUR ligands present a higher affinity when bound to YKL-39 (−7.2 kcal·mol^−1^ in both cases) than their native ligand GlcNAc_2_. The CHI ligand has three hydrogen bonds with Tyr104 (3.61 Å), Asp213 (2.40 Å), and Tyr269 (3.32 Å) and one hydrophobic interaction with Trp360 (3.85 Å), whereas the FUR ligand has three hydrogen bonds with Asp213 (2.97 Å), Tyr104 (3.22 Å), and Phe301 (3.30 Å) and one hydrophobic interaction with Leu105 (3.72 Å). Finally, the highest binding affinity for the YKL-39 enzyme was observed for the CHI/FUR complex (−8.3 kcal·mol^−1^). This complex has four hydrogen bonds with Leu105 (3.72 Å), Asp213 (2.89 Å), Phe301 (3.27 Å), and Trp360 (2.84 Å) and two hydrophobic interactions with Tyr104 (3.77 Å) and Phe301 (3.27 Å). It can be observed that, in the case of the CHI/FUR complex, the cationic CHI creates more interactions with the binding center, while the anionic FUR mostly stabilizes the structure of the complex and is held by the central residue Trp360. As is shown in Figure 2, the CHI and native GlcNAc_2_ ligands occupy the same space in the YKL-39 binding site. The sulfate group of the FUR ligand hinders its deeper binding into the YKL-39 binding site, yet it provides more attraction with cavity amino acids. Finally, the most space-consuming CHI/FUR complex binds shallowly, but its charged character, derived from different functional groups, provides the most attractive interactions with the YKL-39 residues.

### 2.3. Binding of Isolated Ligands and Complexes HA/CHI, HA/CB, and HP/CB to hHyal-1

**Figure 3:**
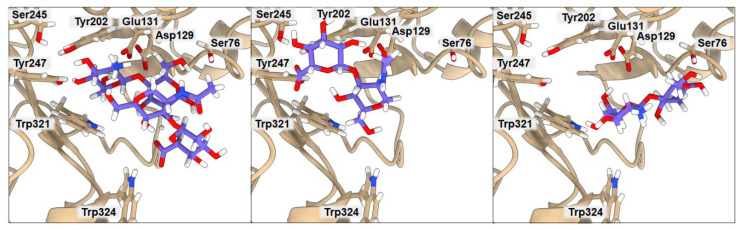
Ribbon representation of the hHyal-1 with docked HA/CHI, HA, and CHI saccharides, from left to right, respectively. Key amino acids that create interactions with ligands are marked in black.

**Figure 4:**
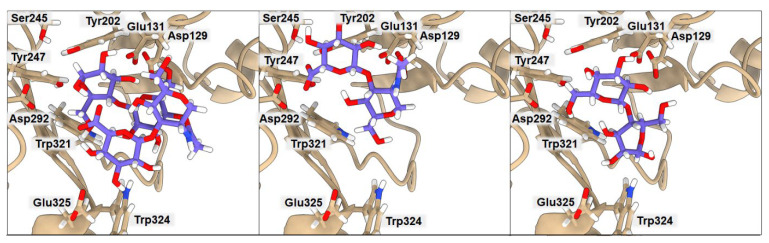
Ribbon representation of the hHyal-1 with docked HA-CB, HA, and CB saccharides, respectively. Key amino acids that create interactions with ligands are marked in black.

**Figure 5:**
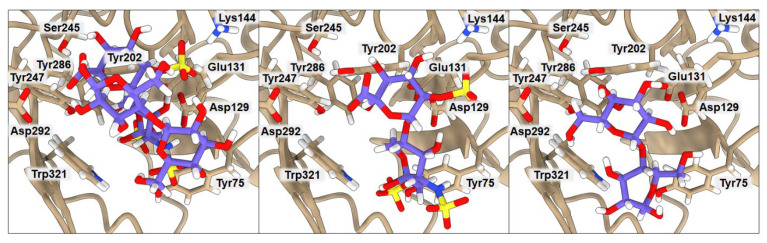
Ribbon representation of hHyal-1 with docked HP-CB, HP, and CB saccharides, respectively. Key amino acids that create interactions with ligands are marked in black.

We examined the binding to hHyal-1 of the set of ligands CHI, HA, CB, and HP and the complexes HA/CHI, HA/CB, and HP/CB. Results are presented in Figure 3, Figure 4, Figure 5 and Table 4, Table 5, Table 6. In all cases, the binding of individual polysaccharides as well as their complexes are dominated by hydrogen bond interactions with the hHYal-1 binding center. 

The anionic HA and HP ligands and the cationic CHI ligand exhibit the same binding affinity (−6.3 kcal·mol^−1^). They all interact with Asp129 (average donor–acceptor distance 3.14 Å), Glu131 (average donor–acceptor distance 2.39 Å), and Trp321 (average donor–acceptor distance 3.21 Å) via hydrogen bonds. In all three cases, Glu131 is closer to the saccharides than Asp129, as it serves as a proton donor for the hydroxyl group for hydrolysis. Asp129 is also a key mechanistic residue, since it neutralizes the positive charge on the nitrogen and facilitates transition-state stabilization. It may stabilize Glu131 via proton sharing and thus can occupy a position farther from the ligand. These two residues have the greatest impact on the final binding affinity value. Interestingly, the low energy structure of HA displays a hydrogen bond network with Tyr202 (donor–acceptor distance 3.02 Å) and Tyr247 (2.99 Å). As shown in Figure 3, the arrangement of those residues is strongly related to Ser245, which does not interact directly with HA but is crucial for the stabilization of the interaction between Tyr202 and Tyr247 observed in the X-ray. A highly sulfated HP glycosaminoglycan required more space, which results in shallower binding into the cleft, with still strong interactions with more external residues such as Tyr75 (2.54 Å) and Lys144 (4.00 Å). Cationic CHI besides Asp129 (3.75 Å), Glu131 (2.91 Å), and Trp321 (3.41 Å), attracts Ser76 (3.19 Å) with hydrogen bonds as well. The binding affinity of CB is almost equal to that of the other ligands (−6.4 kcal·mol^−1^); even though it does not present any ionic character, its eight alcohol (OH) groups can create hydrogen bonds with the residues, Asp129 (3.04 Å), Glu131 (1.87 Å), Tyr202 (2.08 Å), Tyr247 (3.54 Å), and Trp321 (3.19 Å), of hHyal-1. Multiple hydroxyl groups in saccharide ligands tend to find interactions with adjacent Trp321, for HA, HP, CHI, and CB although Trp321 may also create π-stacking or van der Waals hydrophobic attraction.

Non-covalently stabilized complexes bind more strongly than single disaccharides to hHyal-1. They can create more attractive interactions, as observed in the binding affinity equal to −7.2 kcal·mol^−1^ for HA/CHI-hHYal-1 and HA/CB-hHYal-1 or the −7.9 kcal·mol^−1^ computed for HP/CB-hHYal-1. From a structural point of view, most interactions are preserved in the disaccharide complexes, with Asp129 being further than Glu131. In those complexes, hydrogen bond interactions are preponderant. The anionic-cationic HA/CHI complex interacts with Asp129 (3.03 Å), Glu131 (2.52 Å), Tyr202 (2.47 Å), Tyr247 (2.35 Å) via hydrogen bond interactions, and it is stabilized by two π-stacking interactions with Trp321 (3.46 Å) and Trp324 (5.27 Å).

## 3. Computational Methods

In this study, we employed a two-step computational protocol. First, we carried out quantum-mechanical calculations, which allowed us to ascertain the structures and stability of the individual disaccharide ligands and their polyelectrolyte complexes. The three-dimensional structures resulting from the first step were then used in optimized docking calculations to determine their binding affinity and to analyze the key interactions inside YKL-39 or hHyal-1 enzymes.

### 3.1. Quantum-Mechanical Calculations

Single disaccharide ligands and their polyelectrolyte complexes were optimized at the ωB97x-D [13]/def2TZVP [14] level of theory using Gaussian09 [15]. Subsequently, the symmetry-adapted perturbation theory (SAPT) [16] method was employed to study the non-covalent interactions between the saccharides that form a polyelectrolyte complex. This method allows for the decomposition of the overall complex interaction energy, according to Equation (1), into its electrostatic, $E_{elst}^{\left(10\right)}$, exchange, $E_{exch}^{\left(10\right)}$, induction, $E_{ind,resp}^{\left(20\right)}+E_{exch-ind,resp}^{\left(20\right)}+\delta_{HF}^{\left(2\right)}$, and dispersion, $E_{disp}^{\left(20\right)}+E_{exch-disp}^{\left(20\right)}$, components.

(1) $$E_{SAPT0}=E_{elst}^{\left(10\right)}+E_{exch}^{\left(10\right)}+E_{ind,resp}^{\left(20\right)}+E_{exch-ind,resp}^{\left(20\right)}+E_{disp}^{\left(20\right)}+E_{exch-disp}^{\left(20\right)}+\delta_{HF}^{\left(2\right)}$$

The subscript *resp* describes orbital-relaxation effects, while the $\delta_{HF}^{\left(2\right)}$ term corresponds to the higher-order induction effects obtained from the Hartree–Fock (HF) interaction energy. The electrostatic ($\;E_{elst}^{\left(10\right)})$ is a Coulombic charge-clouds-interactions term, the exchange interactions ($E_{exch}^{\left(10\right)}$) is a repulsive force, the induction $\left(E_{ind,resp}^{\left(20\right)}+E_{exch-ind,resp}^{\left(20\right)}+\delta_{HF}^{\left(2\right)}\right)$ covers both polarization and charge-transfer effects, while the dispersion ($E_{disp}^{\left(20\right)}+E_{exch-disp}^{\left(20\right)})$ is an attractive interaction from electron–electron dynamic correlation. The SAPT computations can be very accurate, however, the convergence heavily depends on the size of the system. To study the polyelectrolyte-complex interactions, the SAPT0 [17]/def2TZVP theory level has been used as implemented in the Psi4 [18] program.

### 3.2. Protein-Systems Setup and Interaction Analysis

The initial coordinates of YKL-39 and hHyal-1 were taken from the Protein Data Bank, using PDB entries 4P8V [12] and 2PE4 [19], respectively. Protein backbone and side-chain atoms were checked with ChimeraX [20] and Discovery Studio (version 21.1.0.20298, Dassault Systèmes, Paris, France), while hydrogen atoms were added with the tLeaP module of the Amber program [21]. The GAFF [22] force field was used to prepare the parameters for ligands and complexes. For the case of YKL-39, its native ligand chitooligosaccharide GlcNAc_2_, FUR, CHI, and CHI/FUR complexes were studied; whereas for the hHyal-1 case, CHI, HA, HP, CB, and the complexes HA/CHI, HA/CB, and HP/CB were studied. In all cases, the ligands or polyelectrolyte complexes were docked with the GaudiMM software into their corresponding protein (i.e., YKL-39 or hHyal-1). Key distances and position analysis were evaluated with ChimeraX, DiscoveryStudio, the protein–ligand interaction profiler (PLIP) [23], and LigPlot+ [24]. For the resulting complexes, key-interaction analysis and binding-affinities calculations were performed. The thresholds for key interactions were carefully selected to keep the values as restrictive as possible but also to account for a broad range of interactions: the maximum cut-off for the determination of binding-site atoms was 8.5 Å, the maximum distance of carbon atoms for a hydrophobic interaction was 4.0 Å, the maximum distance between acceptor and donor in hydrogens bonds was 4.1 Å, the minimum angle at the hydrogen-bond donor was 100°, the maximum distance between ring centers for stacking was 7.5 Å, and the maximum deviation from an optimum angle for stacking was 30° [25,26]. The affinities of binding values were computed with the PROtein binDIng enerGY prediction program (PRODIGY) [27]. The Gibbs free energy of binding (ΔG_bind_) is described through the thermodynamic term of Gibbs free energy:

ΔG_bind_ = G_bound_ − G_free_ = RT lnK (2)

where G_bound_ defines the energy of the bound form of the complex, G_free_ corresponds to the sum of the energies of the protein and ligand separately, R is the gas constant, T is the absolute temperature, and K is the equilibrium constant.

### 3.3. Docking Protocol

Most of the current molecular docking protocols are unable to dock ligands that are non-covalently bonded (e.g., hydrogen-bonded) complexes. GaudiMM (Genetic Algorithms with Unrestricted Descriptors for Intuitive Molecular Modeling) is a versatile platform [6] that turned out to be an excellent environment for non-standard-docking tasks. It comprises a multi-objective genetic algorithm that can optimize all the needed variables (objectives) at once and behaves as an evolutionary algorithm for complex molecular problems such as protein–ligand docking. At first, GaudiMM generates an initial population of potential solutions called individuals, which are further evaluated with several objectives. Each of them is assigned a fitness value to find the best results. The best structures are selected by the algorithm and propagated to the next generation. During the next iterations, the initial population of structures evolves to end up with the best set of protein–ligand complexes. For this study, four objectives have been selected: ‘Clashes’ that evaluates steric hindrance and helps to eliminate close contacts between ligand and protein atoms, the AutoDock ‘Vina [28] scoring function’ that is widely used in the literature, an energetic evaluation based on the Amber99SB forcefield for standard protein residues and GAFF-based parametrization [22] for the other atoms of the system, and the ‘HBonds’ descriptor that accounts for protein–ligand hydrogen-bond stabilizations. The latest is the most relevant for the prospect of this work because it helps to displace the GaudiMM conformational exploration towards solutions with good intermolecular hydrogen bond interactions. Out of all the structures received in the docking process, the best possible position had been chosen. The parameters presented such as Clashes, Vina score, energy, and HBonds were taken into consideration to select the set of best structures. Protocols used for running GaudiMM are provided in the Supplementary Material.

**Figure 6:**
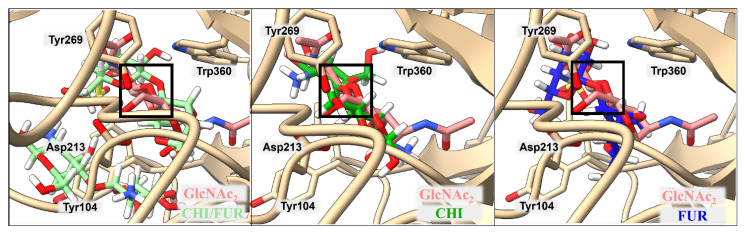
Comparison of CHI/FUR, CHI, and FUR ligand positions obtained by docking with the native GlcNAc_2_ molecule in complex with the 4P8V structure. The glycosidic bond overlap is highlighted in the black box—Cartesian coordinates of atoms in the box are provided in the Supplementary Materials.

The best structures were then compared with the position of the native ligand in the case of 4P8V-bound structures, with particular attention given to the position of the glycosidic bond around the amino acids of the active center (see Figure 6). For ligands bound to 2PE4, where there is no good structure in the Protein Data Bank with a bound ligand, the comparison was made with the position of the HA tetrasaccharide from the published studies, in which the hydrolysis reaction of the peptide bond was investigated [29].

Since GaudiMM simulations in the present form do not allow for a dynamical change of the protonation states, neither of the ligands nor of the protein along the search, protonation states corresponding to the optimal pH conditions for both proteins have been used.

## 4. Conclusions

This work combines quantum-mechanical calculations with atypical protein–ligand-docking experiments to investigate the molecular nature of the interaction of the complexes of disaccharides, as models for polysaccharides, in different key targets.

First, quantum-mechanical calculations were used to show that the disaccharides are capable of forming very strong complexes stabilized by electrostatics and hydrogen bonds. Among the complexes studied here, CHI/FUR and HA/CHI have been shown experimentally to be useful for drug delivery or as an antifouling surface biofilm, and our calculations suggest that two other complexes, HA/CB and HP/CB, are promising drug-encapsulation and delivery candidates. Our results show that the CHI/FUR, HA/CHI, HA/CB, and HP/CB complexes are mostly stabilized by electrostatic interactions, which amount to about 30% of the total complexation energy. A further attraction, approximately 30% of the complexation energy, comes from the induction and dispersion components, which cover polarization, charge transfer, and electron–electron dynamic-correlation effects. The remaining contribution to the total complexation energy is the effects responsible for repulsion. The electrostatic stabilization dominates over the contribution from the hydrogen bonds. Strong non-covalent stabilization makes these complexes excellent candidates for self-assembly smart materials for drug encapsulation and delivery. Their advantage lies also in their biocompatible and biodegradable character [30].

Given that the studied complexes are intended for medical applications, it is worth looking at their enzymatic decomposition in cells, which has not been studied at the molecular level to date. The first stage of evaluating their metabolic consequences is their binding to degrading enzymes and describing the key interactions. This requires specific tools for docking, which include their non-covalent interactions inside the complex stabilization and their non-covalent interactions outside of the complex. Herein, for this nonstandard docking task, we used the GaudiMM engine equipped with a special HBond objective, which drives the conformational exploration towards solutions with hydrogen bonding interactions between the ligand complex and the protein. The separate ligands FUR and CHI and their CHI/FUR complex were docked into YKL-39, which is a specific biomarker for osteoarthritis. Both FUR and CHI interact with the YKL-39 enzyme more strongly than the native GlcNAc_2_ ligand and even more strongly than the separate ligands FUR and CHI bind to their CHI/FUR complex. This complex requires more space than the separate ligands, but at the same time it can create more attractive interactions due to its larger surface. Moreover, its dual anionic/cationic nature allows for finding the optimal orientation inside the enzymatic cavity that leads to the complex of lower energy. The same situation has been observed for the HA/CHI, HA/CB, and HP/CB complexes that were docked to hHYal-1, which is an enzyme involved in hyaluronic acid biodegradation. The complexes, show higher affinities toward the enzyme active site, than the individual ligands. This observation is missing for both the experimental and computational studies, so it requires more focus in the future. Furthermore, our calculations indicate that it is worth considering HP as a potential anionic saccharide to build up the polyelectrolyte complexes, as its sulfate groups create a very stable complex even with neutral cellobiose. This hypothesis is also worth testing more intensively and thoroughly.

## Figures and Tables

**Table 1:** Net charges, number of charged and hydroxyl groups, and number of intermolecular hydrogen bonds of the individual disaccharides and their complexes used in this study.

Disaccharide	Net Charge	# of NH_3_^+^ Groups	# of OH Groups	# of SO_3_^−^ Groups	# of COO^−^ Groups	Complex	Net Charge	# of H-Bonds
HP	−4	0	4	3	1	HP/CB	−4	6
FUR	−1	0	4	1	0	HA/CB	−1	2
HA	−1	0	5	0	1	HA/CHI	+1	3
CB	0	0	8	0	0	CHI/FUR	+1	4
CHI	2	2	6	0	0			

**Table 2:** The contributing components to the complexation energy of complexes computed by using the SAPT0/def2TZVP theory level. Values are reported in kcal·mol^−1^ and as a percentage of the total SAPT0 energy.

Energy Component	HA/CHI		HA/CB		HP/CB		CHI/FUR	
electrostatic	−322.6	27.0%	−46.5	32.6%	−139.7	38.3%	−324.9	29.9%
exchange	541.8	45.4%	49.3	34.6%	124.1	34.1%	508.1	46.7%
induction	−193.9	16.3%	−20.5	14.3%	−60.2	16.5%	−139.7	12.8%
dispersion	−134.5	11.3%	−26.3	18.4%	−40.3	11.1%	−114.8	10.6%
total energy	−109.2	-	−43.9	-	−116.0	-	−71.3	-

**Table 3:** Binding affinity (BA) in kcal·mol^−1^ computed for CHI/FUR, CHI, FUR, and GlcNAc_2_ bound to YKL-39 and key distances in Å between YKL-39 amino acids and ligands. Hydrogen bonds are marked in bold.

	BA	Tyr104	Leu105	Leu210	Asp213	Tyr269	Phe301	Trp360
GlcNAc_2_	−5.7	**3.17**	**3.02**	4.08	**2.59**	3.70	3.91	**3.45**
CHI	−7.2	**3.61**	4.55	4.21	**2.40**	**3.32**	4.86	3.85
FUR	−7.2	**3.22**	3.72	6.81	**2.97**	4.84	**3.30**	4.02
CHI/FUR	−8.3	3.77	**2.65**	6.23	**2.89**	4.29	**3.27**	**2.84**

**Table 4:** Binding affinity (BA) computed for HA/CHI, HA, and CHI bound to hHyal-1, and representation of key distances between hHyal-1 amino acids and ligands. Values are reported in kcal·mol^−1^ and Å for BA and distances, respectively. Hydrogen bonds are marked in bold.

	BA	Ser76	Asp129	Glu131	Tyr202	Tyr247	Trp321	Trp324
HA	−6.3	7.93	**2.20**	**2.05**	**3.02**	**2.99**	**3.53**	**3.46**
CHI	−6.3	**3.19**	**3.75**	**2.91**	8.15	6.16	**3.41**	5.30
HA/CHI	−7.2	4.71	**3.03**	**2.52**	**2.47**	**2.35**	3.46	5.27

**Table 5:** Binding affinity (BA) in kcal·mol^−1^ computed for HA-CB, HA, and CB bound to hHyal-1 and key distances in Å between Hyal-1 amino acids and ligands. Hydrogen bonds are marked in bold.

	BA	Asp129	Glu131	Tyr202	Tyr247	Asp292	Trp321	Trp324	Glu325
HA	−6.3	**2.20**	**2.05**	**3.02**	**2.99**	**2.96**	**3.53**	**3.46**	6.37
CB	−6.4	**3.04**	**1.87**	**2.08**	**3.54**	**2.84**	**3.19**	**2.60**	4.29
HA/CB	−7.2	**2.87**	**2.60**	**2.54**	**3.27**	**3.86**	4.15	**3.68**	**3.15**

**Table 6:** Binding affinity (BA) in kcal·mol^−1^ computed for HP-CB, HP, and CB bound to hHyal-1 and key distances in Å between Hyal-1 amino acids and ligands. Hydrogen bonds are marked in bold.

	BA	Tyr75	Asp129	Glu131	Lys144	Tyr202	Tyr247	Tyr286	Asp292	Trp321
HP	−6.3	**2.54**	**3.46**	**2.20**	**4.00**	5.79	6.32	8.56	5.01	**3.23**
CB	−6.4	4.04	**3.04**	**1.87**	**5.30**	**2.08**	**3.54**	8.58	**2.84**	**3.19**
HP/CB	−7.9	**3.39**	**2.67**	**1.91**	**3.61**	**3.09**	**3.72**	**2.52**	**3.03**	**2.91**

## Data Availability

Not applicable.

## Supplementary Materials

The supporting information can be downloaded at: https://www.mdpi.com/1422-0067/23/14/7705/s1.
