# Supplementary material for: Influence of Association on Binding of Disaccharides to YKL-39 and hHyal-1 Enzymes
Source: Int J Mol Sci. 2022 Jul 12;23(14):7705. doi: 10.3390/ijms23147705 (PMC9317946; doi:10.3390/ijms23147705)
Supplement: Supplementary file 1 [file ijms-23-07705-s001.zip › ijms-1749906-supplementary.pdf]

Supporting Materials: Influence of association on binding of disaccharides to YKL-39 and hHyal-1 enzymes

Agnieszka Krzemińska,[1] José-Emilio Sánchez-Aparicio,[2] Jean-Didier Maréchal,[2*] Agata Paneth,[3] and Piotr Paneth[1*]

1. International Center for Research on Innovative Biobased Materials (ICRI-BioM) - International Research Agenda, Lodz University of Technology, Żeromskiego 116, 90-924 Łódź, Poland

2. Insilichem, Departament de Química, Facultat de Ciències, Universitat Autònoma de Barcelona, 08193 Bellaterra (Barcelona), Spain

3. Faculty of Pharmacy, Medical University of Lublin, Chodźki 4a, 20-210 Lublin, Poland

Table S1. Cartesian coordinates for CHI/FUR complex suitable for 4P8V PDB structure.

C1	-25.215	31.239	4.375
C2	-24.288	33.234	3.147
C3	-25.512	32.671	3.908
C4	-26.348	30.510	5.112
O5	-27.080	31.323	5.996
S6	-27.084	32.814	2.973
O7	-27.404	31.508	2.334
O8	-28.075	33.075	4.089
O9	-26.958	33.926	2.037
H10	-25.682	33.328	4.775
H11	-26.988	30.059	4.332
H12	-25.921	29.688	5.706
H13	-27.404	32.107	5.499
C14	-23.782	30.857	2.533
O15	-23.628	30.051	1.403
C16	-25.231	26.306	0.227
O17	-25.703	25.070	0.625
H18	-25.235	24.778	1.422
C19	-24.073	28.729	1.413
C20	-25.512	28.645	0.873
O21	-25.965	27.290	0.981
C22	-23.727	26.495	0.383
C23	-23.394	27.985	0.266
C24	-25.266	29.060	-0.576
O25	-23.998	28.516	-0.905
H26	-26.222	29.283	1.417
H27	-23.217	25.915	-0.402
H28	-23.398	26.108	1.364
H29	-22.312	28.169	0.219
H30	-25.248	30.160	-0.660
H31	-26.031	28.667	-1.270
H32	-25.511	26.406	-0.835
H33	-23.959	28.248	2.397
O34	-24.877	30.416	3.282
H35	-22.862	30.802	3.157
H36	-24.344	31.328	5.057
C37	-23.939	32.288	2.001
O38	-22.748	32.702	1.409
H39	-24.779	32.275	1.276
H40	-23.446	33.200	3.863
O41	-24.415	34.562	2.787
H42	-22.573	32.129	0.648
H43	-25.212	34.662	2.234
H44	-31.012	32.310	5.620
C45	-29.891	27.427	-1.417
C46	-29.051	26.506	-0.539
C47	-29.770	26.332	0.784
C48	-30.023	27.703	1.411
C49	-30.170	28.740	-0.664
O50	-30.833	28.422	0.551
H51	-30.862	26.936	-1.599
O52	-29.182	27.611	-2.609
H53	-28.076	27.010	-0.357
O54	-28.851	25.237	-1.083

N55	-28.971	25.469	1.689
H56	-30.737	25.834	0.622
C57	-31.013	29.705	-1.480
H58	-29.212	29.245	-0.428
O59	-31.941	28.975	-2.241
H60	-29.805	27.888	-3.296
H61	-28.553	25.344	-2.001
H62	-29.514	25.240	2.497
H63	-30.335	30.292	-2.130
H64	-31.498	30.402	-0.772
H65	-32.554	29.580	-2.679
C66	-30.258	28.364	3.689
C67	-30.667	29.831	3.482
C68	-30.386	30.537	4.803
C69	-31.021	29.891	6.042
C70	-30.902	27.792	4.958
O71	-30.549	28.571	6.080
H72	-29.155	28.338	3.808
O73	-30.637	27.512	2.631
H74	-31.746	29.848	3.245
O75	-30.016	30.481	2.437
N76	-30.675	31.994	4.733
H77	-29.306	30.476	4.972
O78	-30.672	30.570	7.174
C79	-30.446	26.373	5.272
H80	-32.004	27.792	4.833
O81	-30.983	25.912	6.465
H82	-29.056	30.588	2.654
H84	-29.333	26.359	5.274
H85	-30.805	25.699	4.476
H86	-30.802	26.574	7.151
H87	-29.052	28.229	1.550
H88	-32.128	29.897	5.983
H89	-29.687	30.575	7.264
H90	-28.717	24.629	1.210
H91	-31.370	32.163	4.034

Table S2. Cartesian coordinates for CHI ligand suitable for 4P8V PDB structure.

H1	-25.211	26.301	-1.840
C2	-25.149	34.120	1.833
C3	-25.748	33.453	3.065
C4	-25.009	32.151	3.300
C5	-25.086	31.285	2.044
C6	-25.186	33.140	0.647
O7	-24.467	31.972	1.015
H8	-24.088	34.344	2.045
O9	-25.880	35.291	1.608
H10	-26.815	33.227	2.841
O11	-25.643	34.218	4.227
N12	-25.581	31.444	4.473
H13	-23.951	32.355	3.523
C14	-24.594	33.739	-0.617
H15	-26.237	32.861	0.428
O16	-23.520	34.577	-0.269

H17	-25.340	35.892	1.075
H18	-25.958	35.114	4.029
H19	-25.000	30.666	4.710
H20	-25.391	34.306	-1.138
H21	-24.288	32.902	-1.269
H22	-23.067	34.875	-1.069
C23	-25.014	28.919	1.764
C24	-24.961	28.851	0.229
C25	-25.401	27.439	-0.143
C26	-24.632	26.306	0.548
C27	-24.254	27.743	2.389
O28	-24.780	26.518	1.927
H29	-26.079	28.854	2.066
O30	-24.445	30.092	2.301
H31	-23.916	29.036	-0.080
O32	-25.747	29.794	-0.428
N33	-25.463	27.243	-1.618
H34	-26.436	27.330	0.195
O35	-25.131	25.090	0.182
C36	-24.362	27.700	3.908
H37	-23.182	27.819	2.111
O38	-23.733	26.583	4.438
H39	-26.705	29.602	-0.268
H41	-25.441	27.747	4.179
H42	-23.855	28.584	4.326
H43	-24.064	25.801	3.969
H44	-26.153	31.098	1.789
H45	-23.557	26.316	0.275
H46	-26.090	25.042	0.421
H47	-25.630	32.072	5.250
H48	-24.828	27.873	-2.065

Table S3. Cartesian coordinates for FUR ligand suitable for 4P8V PDB structure.

C1	-26.896	32.277	3.391
C2	-24.720	33.541	3.293
C3	-26.231	33.591	2.961
C4	-28.397	32.140	3.091
O5	-29.137	33.319	3.294
S6	-26.582	34.043	1.219
O7	-26.876	32.812	0.434
O8	-27.855	34.850	1.368
O9	-25.456	34.823	0.713
H10	-26.654	34.438	3.524
H11	-28.476	31.771	2.052
H12	-28.827	31.371	3.750
H13	-28.718	34.041	2.774
C14	-24.907	31.056	3.008
O15	-24.380	30.024	2.232
C16	-26.591	26.739	0.418
O17	-27.748	26.013	0.630
H18	-27.932	25.954	1.579
C19	-25.081	28.926	1.666
C20	-25.909	29.084	0.377
O21	-26.975	28.127	0.396

C22	-25.483	26.485	1.432
C23	-24.430	27.590	1.312
C24	-24.840	28.740	-0.658
O25	-24.047	27.735	-0.048
H26	-26.352	30.083	0.262
H27	-25.040	25.497	1.229
H28	-25.903	26.469	2.454
H29	-23.533	27.380	1.911
H30	-24.228	29.629	-0.885
H31	-25.265	28.356	-1.602
H32	-26.243	26.461	-0.591
H33	-25.706	28.900	2.572
O34	-26.280	31.174	2.766
H35	-24.742	30.834	4.087
H36	-26.745	32.216	4.488
C37	-24.108	32.309	2.628
O38	-22.787	32.169	3.046
H39	-24.183	32.422	1.526
H40	-24.655	33.384	4.386
O41	-24.051	34.725	3.048
H42	-22.396	31.423	2.571
H43	-24.169	34.963	2.110

Protocol S1.Docking protocole to 4P8V. PDB structure


```yaml
_path: 4p8v_fur.yaml
ga:
    cx_eta: 5
    cx_pb: 0.5
    generations: 100
    lambda_: 3
    mu: 1.0
    mut_eta: 5
    mut_indpb: 1.0
    mut_pb: 0.5
    population: 100
genes:
-   module: gaudi.genes.molecule
    name: Ligand0
    path: ./lig.mol2
-   module: gaudi.genes.molecule
    name: Protein
    path: ./protein.mol2
-   center:
    - -24.118
    - 30.171
    - 1.834
    interpolation: 0.5
    module: gaudi.genes.search
```

```
    name: Search0
    precision: 5
    radius: 8.0
    rotate: true
    target: Ligand0
-   library: dunbrack
    module: gaudi.genes.rotamers
    name: Rotamers
    residues:
    - Protein/213
    - Protein/269
    - Protein/360
objectives:
-   module: gaudi.objectives.hbonds
    name: HBonds
    only_probes: true
    probes:
    - Ligand0
    weight: 1.0
-   module: gaudi.objectives.energy
    name: Energy
    weight: -1.0
    targets: [Protein, Ligand0]
    forcefields: [amber99sbildn.xml]
    parameters: [[lig.mol2, lig.frcmod]]
-   module: gaudi.objectives.contacts
    name: Clashes
    probes:
    - Ligand0
    radius: 5.0
    weight: -1.0
    which: clashes
-   ligand: Ligand0
    module: gaudi.objectives.vina
    name: Vina0
    receptor: Protein
    weight: -1.0
output:
    check_every: 0
    compress: true
    history: false
    name: lig_4p8v
    pareto: false
    path: lig_4p8v
    precision: 3
    prompt_on_exception: true
    verbose: true
similarity:
    args:
```

```yaml
    -   - Ligand0
    - 2.5
    kwargs: {}
    module: gaudi.similarity.rmsd
```

Protocol S2. Docking protocole to 2PE4.PDB structure


```yaml
_path: 2pe4_ha.yaml
ga:
    cx_eta: 5
    cx_pb: 0.5
    generations: 100
    lambda_: 3
    mu: 1.0
    mut_eta: 5
    mut_indpb: 1.0
    mut_pb: 0.5
    population: 100
genes:
-   module: gaudi.genes.molecule
    name: Ligand0
    path: ./lig.mol2
-   module: gaudi.genes.molecule
    name: Protein
    path: ./protein.mol2
-   center:
    - 37.41
    - -22.557
    - -17.999
    interpolation: 0.5
    module: gaudi.genes.search
    name: Search0
    precision: 5
    radius: 8.0
    rotate: true
    target: Ligand0
-   library: dunbrack
    module: gaudi.genes.rotamers
    name: Rotamers
    residues:
    - Protein/129
    - Protein/131
    - Protein/202
objectives:
-   module: gaudi.objectives.hbonds
    name: HBonds
```

```yaml
    only_probes: true
    probes:
    - Ligand0
    weight: 1.0
-   forcefields:
    - amber99sbildn.xml
    module: gaudi.objectives.energy
    name: Energy
    parameters:
    -   - lig_gaff.mol2
        - lig.frcmod
    targets:
    - Protein
    - Ligand0
    weight: -1.0
-   module: gaudi.objectives.contacts
    name: Clashes
    probes:
    - Ligand0
    radius: 5.0
    weight: -1.0
    which: clashes
-   ligand: Ligand0
    module: gaudi.objectives.vina
    name: Vina0
    receptor: Protein
    weight: -1.0
output:
    check_every: 0
    compress: true
    history: false
    name: lig_2pe4
    pareto: false
    path: lig_2pe4
    precision: 3
    prompt_on_exception: true
    verbose: true
similarity:
    args:
    -   - Ligand0
    - 2.5
    kwargs: {}
    module: gaudi.similarity.rmsd
```
